# Infectious hematopoietic necrosis virus specialization in a multihost salmonid system

**DOI:** 10.1111/eva.12931

**Published:** 2020-02-28

**Authors:** David J. Páez, Shannon L. LaDeau, Rachel Breyta, Gael Kurath, Kerry A. Naish, Paige F. B. Ferguson

**Affiliations:** ^1^ Department of Biological Sciences The University of Alabama Tuscaloosa Alabama; ^2^ Cary Institute of Ecosystem Studies Millbrook New York; ^3^ U.S. Geological Survey, Western Fisheries Research Center Seattle Washington; ^4^ School of Aquatic and Fishery Sciences University of Washington Seattle Washington

**Keywords:** Bayesian models, fish diseases, salmon hatcheries, specialist/generalist trade‐offs, transmission

## Abstract

Many pathogens interact and evolve in communities where more than one host species is present, yet our understanding of host–pathogen specialization is mostly informed by laboratory studies with single species. Managing diseases in the wild, however, requires understanding how host–pathogen specialization affects hosts in diverse communities. Juvenile salmonid mortality in hatcheries caused by infectious hematopoietic necrosis virus (IHNV) has important implications for salmonid conservation programs. Here, we evaluate evidence for IHNV specialization on three salmonid hosts and assess how this influences intra‐ and interspecific transmission in hatchery‐reared salmonids. We expect that while more generalist viral lineages should pose an equal risk of infection across host types, viral specialization will increase intraspecific transmission. We used Bayesian models and data from 24 hatcheries in the Columbia River Basin to reconstruct the exposure history of hatcheries with two IHNV lineages, MD and UC, allowing us to estimate the probability of juvenile infection with these lineages in three salmonid host types. Our results show that lineage MD is specialized on steelhead trout and perhaps rainbow trout (both *Oncorhynchus mykiss*), whereas lineage UC displayed a generalist phenotype across steelhead trout, rainbow trout, and Chinook salmon. Furthermore, our results suggest the presence of specialist–generalist trade‐offs because, while lineage UC had moderate probabilities of infection across host types, lineage MD had a small probability of infection in its nonadapted host type, Chinook salmon. Thus, in addition to quantifying probabilities of infection of socially and economically important salmonid hosts with different IHNV lineages, our results provide insights into the trade‐offs that viral lineages incur in multihost communities. Our results suggest that knowledge of the specialist/generalist strategies of circulating viral lineages could be useful in salmonid conservation programs to control disease.

## 1 INTRODUCTION

The degree to which pathogens evolve to use different host species can reflect varying levels of pathogen specialization (Dybdahl & Storfer, 2003). Theoretical and experimental work has demonstrated how specialization can have unexpected consequences on epidemic outcomes, transmission rates, and the evolution of virulence (Auld, Searle, & Duffy, 2017; Gandon, 2004; Lievens, Perreau, Agnew, Michalakis, & Lenormand, 2018; Rigaud, Perrot‐Minnot, & Brown, 2010; Woolhouse, Taylor, & Haydon, 2001). However, there is less evidence for how specialist and generalist virus lineages coexist and circulate in nature (Real, Russell, Waller, Smith, & Childs, 2005), despite extensive work under laboratory conditions (but see Fallon, Bermingham, & Ricklefs, 2005; McCoy, Léger, & Dietrich, 2013, for field examples). Determining whether specialization occurs across the distribution of hosts may allow us to better evaluate whether specialized host–pathogen associations are important for the management of disease in wildlife. Previous work in crop plants, for example, has demonstrated that the severity of disease resulting from specialized pathogens in monocultures can be reduced by increasing cultivar diversity (Cox, Garrett, & Bockus, 2005; Mitchell, Tilman, & Groth, 2002). In the current study, we use data‐driven transmission models to examine patterns of exposure and infection by distinct lineages of infectious hematopoietic necrosis virus (IHNV), and evaluate each lineage's generalist/specialist phenotype across hatchery‐reared salmonid populations in the Columbia River Basin of Washington and Oregon, USA.

Salmonids are of great economic and cultural importance (Rushton, 2011). However, due to a multitude of factors that include habitat changes by humans, exploitation, and habitat pollution, salmon stocks have been declining throughout their native range in the Northern Hemisphere (Gustafson et al., 2007; Quinn, 2018). In response to decreasing population sizes, hatcheries, which are facilities where juvenile salmon are bred from adults and reared for a period of time before being released back into the rivers, have been established throughout the Pacific Northwest of North America. Despite the implementation of extensive biosecurity measures in hatcheries to preserve fish health (McDaniel et al., 1994), juvenile fish still contract diseases caused by infectious pathogens, such as IHNV (Gaest et al., 2011; Naish et al., 2008). IHNV outbreaks can cause high juvenile mortality and result in the release of large numbers of virus particles into water sources (Naish et al., 2008).

The first documented IHNV epizootics in hatcheries in the Columbia River Basin occurred in the 1950s. IHNV has been detected frequently thereafter both in the Basin and in most Pacific watersheds of North America (Bootland & Leong, 2011; Wolf, 1988). The pathology of IHNV includes acute necrosis of the kidney and spleen, although infection and recovery can sometimes occur without clinical signs of disease (Bootland & Leong, 2011). IHNV can be transmitted vertically (Mulcahy & Bauersfeld, 1983); however, the majority of transmission is considered to occur horizontally through the water column (Bootland & Leong, 2011; Mulcahy, Pascho, & Jenes, 1983; Pilcher & Fryer, 1980), as supported by observations that returning adult spawners are an important source of IHNV exposure to hatchery‐reared juveniles (Breyta, Samson, Blair, Black, & Kurath, 2016; Ferguson, Breyta, Brito, Kurath, & LaDeau, 2018).

Infectious hematopoietic necrosis virus is a rhabdovirus that displays negligible recombination, as typical of negative sense viruses. IHNV genetic diversity is well defined, with three major North American genogroups (U, M, and L) further resolved into as many as 13 lineages, distributed across the Pacific coast from California to Alaska (Black, Breyta, Bedford, & Kurath, 2016; Breyta, Black, Kaufman, & Kurath, 2016; Kelley, Bendorf, Yun, Kurath, & Hedrick, 2007; Kurath et al., 2003; Troyer & Kurath, 2003). These lineages have been observed to infect different salmonid hosts at different frequencies, suggesting that lineage diversity could be partly driven by the evolution of host specificity (Black et al., 2016; Breyta, Black, et al., 2016; Garver, Troyer, & Kurath, 2003; Kurath et al., 2003). The L, U, and M viral genogroups are most often observed in Chinook salmon (*Oncorhynchus tshawytscha*, in the southern distribution of the species), sockeye salmon (*Oncorhynchus nerka*), and steelhead and rainbow trout (both *Oncorhynchus mykiss*), respectively (Breyta, Black, et al., 2016; Kurath, 2012; Kurath et al., 2003). An exception to this pattern occurs in the Columbia River Basin, where evidence suggests that a recently evolved lineage, UC (detected in the 1970s), within the U genogroup is frequently observed in several salmonid species (Black et al., 2016; Breyta, Black, et al., 2016). This multihost UC lineage arose from the ancestral sockeye‐specific U genogroup (Black et al., 2016). Lineage UC co‐occurs with two additional lineages in the Columbia River Basin, MD and UP, that are strongly supported by phylogenetic analyses. The genetic divergence between these lineages, measured as mean percent difference, is 3.3% between MD and UC, 3.3% between MC and UP, and 1.1% between UC and UP (Breyta, Black, et al., 2016). Lineage MD evolved within the M genogroup and is thought to be specialized on steelhead and rainbow trout (anadromous and freshwater life‐history types of *O. mykiss*, respectively), based on the high infection frequency and high virulence observed in these hosts (Breyta, Black, et al., 2016; Garver, Batts, & Kurath, 2006; Garver et al., 2003; Troyer & Kurath, 2003; Troyer, LaPatra, & Kurath, 2000). Lineage UP also evolved within the U genogroup, but contrary to lineages MD and UC, the observed frequency of lineage UP is low across the Columbia River Basin (Black et al., 2016).

Although the observed frequency of infection with an IHNV lineage varies across different salmonid hosts (Black et al., 2016; Breyta, Black, et al., 2016; Garver et al., 2003), we do not know whether this results from specialist/generalist host–pathogen associations. Indeed, additional factors that could generate such variation include environmental stochasticity (e.g., random fluctuation in pathogen incidence), host species geographic distributions, pathogen evolution (e.g., older lineages with higher prevalence than newly evolved lineages), husbandry practices that vary for different host types, or variation in disease management practices across hatcheries. Furthermore, measuring IHNV infection and genotyping virus isolates to find lineage identity is logistically challenging, so there is inevitable variation in the extent of data coverage across salmonid hatcheries (Ferguson et al., 2018). Variation in the observed frequency of infection between lineages could thus also arise from inconsistent sampling methods that result in missing data.

Previous work that accounted for variation in missing data showed that exposure to IHNV‐infected adults returning from the ocean to spawn in the Columbia River Basin was the most likely source of IHNV exposure for hatchery juveniles, with juvenile–juvenile exposure across cohorts occurring less frequently but having a higher probability of infection given exposure (Breyta, Brito, Ferguson, et al., 2017; Ferguson et al., 2018). However, these results assumed a homogeneous composition of hosts and IHNV lineages across hatcheries, making it difficult to determine whether infection dynamics depend on specific host–pathogen associations. In this study, we aim to estimate the probability of infection of different salmonid hosts given exposure to different IHNV lineages to understand the role that IHNV specialization plays in the infection dynamics of hatcheries in the lower Columbia River Basin.

Specifically, we first test for differences in the probability of infection given exposure to the three IHNV lineages, MD, UC, and UP, assuming a homogeneous host identity (using a lineage model). Then, by assuming a homogeneous lineage identity, we test for differences in the probability of infection of different hosts (steelhead trout, rainbow trout, and Chinook salmon) given that exposure occurs by the same host compared to when it occurs by other hosts (using a host model). Lastly, we test whether the probability of infection of the three host types depends on the lineage to which they are exposed (using a host‐by‐lineage model). Given exposure to pathogens, specialized lineages should pose a higher risk of infection to the host to which they are specialized, whereas generalist pathogens should pose an equal risk of infection across hosts. Therefore, given exposure, differences in a lineage's probability of infection across different hosts should indicate whether pathogen lineages are specialists or generalists.

## 2 MATERIALS AND METHODS

### 2.1 Data structure

**Figure 1:**
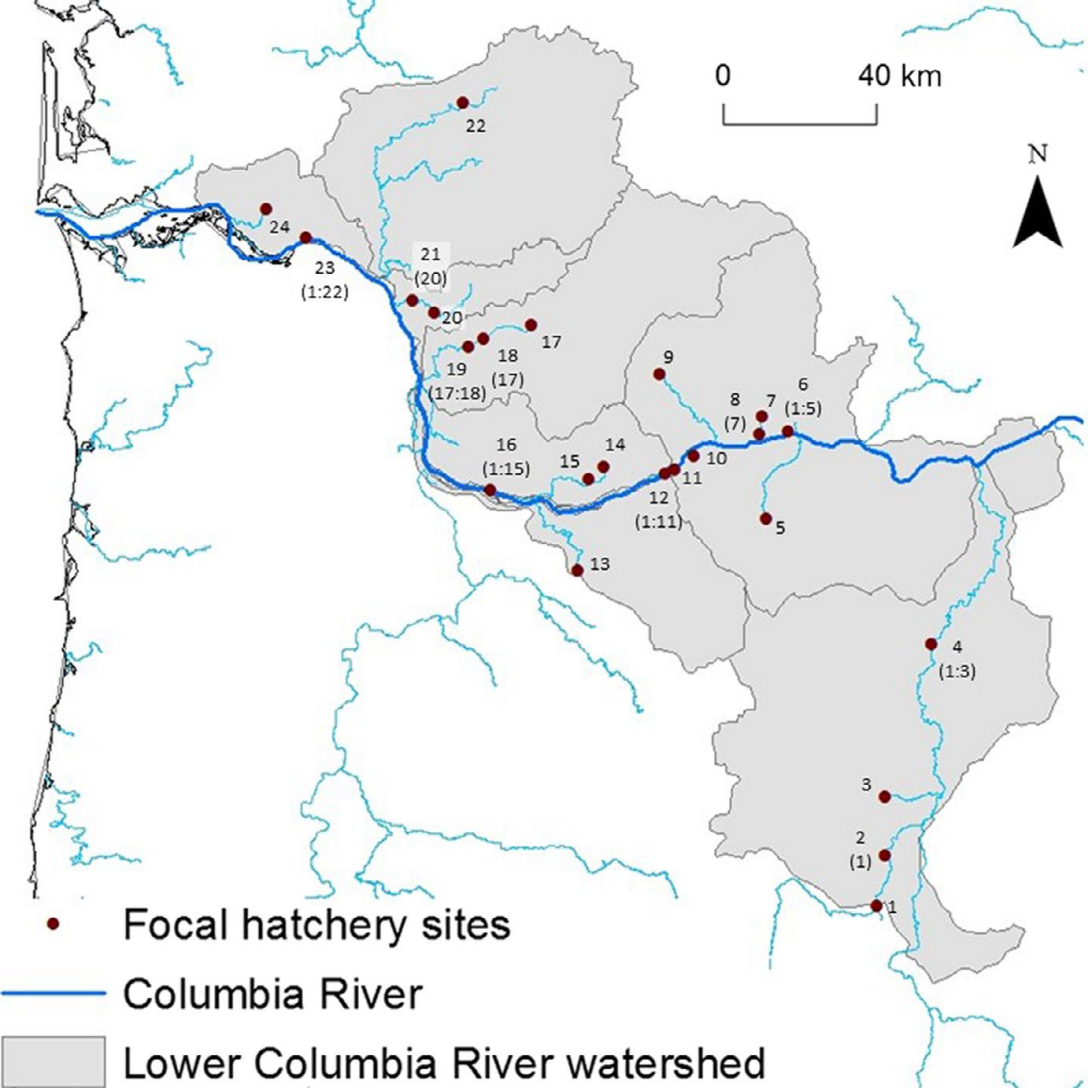
Map showing the location of the 24 sites (red dots). Each site is labeled with a number. Numbers in parentheses are associated with downstream sites and indicate the label of the upstream sites with potential to act as a source of IHNV (e.g., site 4 is downstream of sites 1–3). Ten of the 24 sites were downstream of other sites

We developed three models to estimate the probability of juvenile infection given exposure to IHNV across 13 cohorts (sampled from 2000 to 2012) at 24 hatchery sites located in the Columbia River for a total of 312 cohort‐sites (Figure 1). These cohort‐sites are a subset of a larger data set documenting IHNV incidence described in Breyta, Brito, Kurath, and LaDeau (2017). A cohort‐site refers to the juvenile or adult fish present at a site (i.e., hatchery) at a particular time. A juvenile cohort consisted of fish tested for IHNV between June 1 of any given year and May 31 of the following year, whereas an adult cohort comprised the adult fish tested for IHNV between August 1 of any given year and July 31 of the following year (Ferguson et al., 2018). We assumed that a cohort‐site was IHNV‐positive if any juvenile test from that cohort‐site was positive (Breyta, Brito, Kurath, et al., 2017). Fish were diagnosed IHNV‐positive or IHNV‐negative by following standard protocols of cell culture and RT‐PCR (AFS‐FHS, 2014). Genotyping of positive records to identify lineages was conducted by Sanger sequencing of the midG region (303 nucleotides) and Bayesian phylogenetic analyses, as described in previous work (Breyta, Brito, Ferguson, et al., 2017; Emmenegger & Kurath, 2002; Kurath et al., 2003).

**Figure 2:**
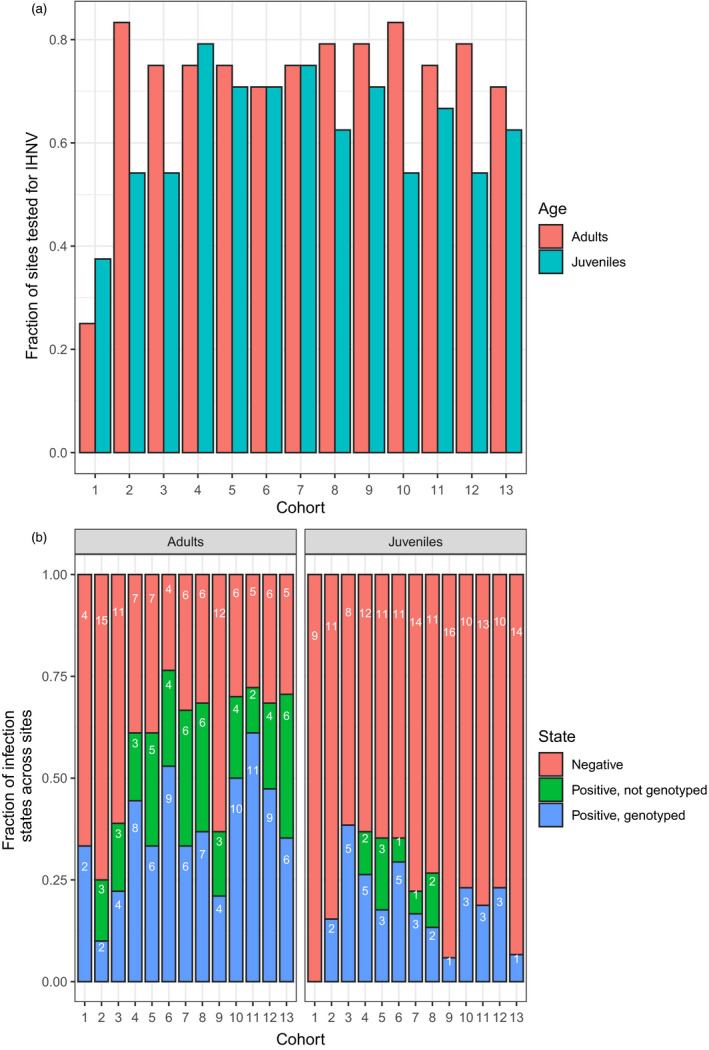
(a) Data coverage across sites for each cohort. The bar height represents the number of sites tested in a cohort divided by the number of sites, which is always 24. (b) Infection states of sites in each cohort. Fraction indicates the count of the infection state (white‐font numbers) divided by the total number of sites that were tested in each cohort

Missing test records (i.e., no positive/negative IHNV tests) in juveniles and adults occurred in 37.5% and 27% of cohort‐sites, respectively (Figure 2a). Specifically, neither adults nor juveniles were tested for IHNV in 11.5% of cohort‐sites; adults but not juveniles were tested in 26% of cohort‐sites; and juveniles but not adults were tested in 15.7% of cohort‐sites. Thus, both adults and juveniles were tested in 47% of cohort‐sites. Furthermore, the IHNV lineage identity was missing if no positive samples at a cohort‐site were genotyped. In cohort‐sites where some positive samples were genotyped, we assumed that the nongenotyped samples were the same genotype(s) as the genotyped sample(s). Across adults and juveniles, 37% and 20% of cohort‐sites that tested positive were not genotyped, respectively (Figure 2b).

### 2.2 IHNV exposure mechanisms

We assumed that juveniles could be exposed to IHNV via three nonmutually exclusive routes (Ferguson et al., 2018). Route 1 assumed that exposure occurred if IHNV‐positive juveniles were detected at the same hatchery in the previous cohort. Exposure via Route 2 occurred if IHNV‐positive juveniles were found in hatcheries located upstream from the focal hatchery (Figure 1) in the current or previous cohort. Route 3 occurred if IHNV‐positive adults were detected at the same hatchery or in an upstream hatchery in the previous cohort or the current cohort (Breyta, Black, et al., 2016; Ferguson et al., 2018) (Figure 1). Inferring these exposure routes from the data depends on knowing the infection status of adults or juveniles in the previous cohort. Thus, it was not possible to determine exposure routes for juveniles in the first cohort. Similarly, we did not model exposure of adult fish because we do not know whether adults were infected at their hatchery of origin, or after release, during their river and ocean migrations. Nevertheless, we estimated the probability of infection for juveniles in the first cohort and for adults across all cohorts without making any assumptions about exposure mechanisms, as described below.

### 2.3 General model flow

The first step in fitting the three models to the data was to estimate the probability of infection of adults and first‐cohort juveniles (see Figure S1 for model algorithms). For these two groups, the probability of infection at each cohort was estimated using Bernoulli trials with constant probabilities of infection and vague prior distributions (Beta(1, 1); see Table A1 for a description of all model parameters). The second step, which was model‐specific, was identifying the routes of IHNV exposure (for juvenile cohort‐sites >1) and estimating the probability of infection given that exposure to IHNV occurred by at least one of the described routes (Figure S2).

Within both of these steps, we incorporated procedures to infer probabilities of infection when there were missing data due to a lack of IHNV testing or genotyping of positive tests. In cases where missing values occurred because of a lack of testing, models inferred the unknown infection state (i.e., positive or negative) using a multiple imputation procedure (described in Carrigan, Barnett, Dobson, & Mishra, 2007; Pettitt, Tran, Haynes, & Hay, 2006; Rubin, 2004) and informative priors for the probability of infection given no testing. These prior distributions were built using previous knowledge of the IHNV‐positive testing rate in adults and juveniles which were obtained from the routine IHNV testing conducted by agencies to monitor adult and juvenile fish health (Ferguson et al., 2018). The mean incidence of IHNV‐positive tests in adults was 0.26, with a standard deviation of 0.02. In juveniles, the incidence and standard deviation were 0.007 and 0.01, respectively. To build the prior distributions, we used these values to calculate shape parameters for beta distributions, resulting in priors Beta(122, 354) and Beta(0.5, 68) for adults and juveniles, respectively (see also Ferguson et al., 2018). This prior‐informed imputation procedure then allowed us to infer whether untested cohort‐sites were IHNV‐positive or IHNV‐negative.

The imputation of missing genotypes of positive tests followed a similar logic, using the lineage‐specific positive rate across all positive tests to build informative prior distributions and a multiple imputation procedure. We calculated the mean lineage‐specific infection rate as the number of cohort‐sites that were positive with each lineage divided by the total number of positive cohort‐sites that were genotyped. The standard deviation, *SD*, was then calculated as

$$SD=\sqrt{\frac{p(1-p)}{n}},$$

where *p* is the mean lineage‐specific positive rate and *n* is the number of samples. We then used these values to calculate shape parameters of a beta distribution, which we used as priors (see Table A1 for specific values).

Posterior distributions of parameter values were generated using Markov chain Monte Carlo sampling routines implemented in R 3.4.3 and OpenBUGS (using the R2OpenBUGS package (Sturtz, Ligges, & Gelman, 2005)). In all models, we ran three chains of 1.1 ×10^5^ iterations, discarding the first 10^4^ steps. Chains were thinned by keeping every third step. Summary statistics of posterior distributions (medians and 95% credible intervals [CI]) were calculated after verifying that values of the Gelman–Rubin potential scale reduction factor (R‐hat), which suggests chain convergence, were between 1 and 1.02 (Brooks & Gelman, 1998).

### 2.4 Model testing

We used model simulations to identify estimation biases and to quantify the levels of parameter uncertainty under different scenarios of missing data. Thus, we created three scenarios that mimicked different levels of data completeness: no missing data (Scenario 1), missing data due to no testing but complete genotyping of positive tests (Scenario 2), and missing data due to no testing and incomplete genotyping of positive tests (Scenario 3; see more details in Appendix S1: Section 1). For each model and data scenario, we then simulated data based on a set of true parameter values. By fitting the models to these data as described above, we determined how accurate and precise the models were in recovering the true parameter values.

Model simulations provided context for evaluating results from the model fit to the real data. The complete results of the model simulations are presented in Appendix S1, but we highlight key findings, particularly of Scenario 3, in the results.

### 2.5 Lineage model

In the lineage model, we estimated the probability of infection with different IHNV lineages (MD, UC, or UP) across the cohort‐sites, without including the effects of host identity. This model allowed us to determine whether the probability of infection with the three lineages can be precisely estimated to help interpret the final host‐by‐lineage model (which contains a large number of parameters). We expect to obtain precise estimates of the probabilities of infection with lineages UC and MD and low precision for the probability of infection with lineage UP. This is because lineage UC is often detected across several salmonid hosts and lineage MD is frequently detected in steelhead and rainbow trout. By contrast, lineage UP is only rarely observed in the studied hatcheries.

If the cohort‐site was not tested or was positive but not genotyped, then the probability of juvenile infection given no test (*ϵ*) and the probabilities of infection with a particular lineage given no genotyping of positive tests (*µ*_1_, *µ*_2_ and *µ*_3_) were inferred as described above under “Section 2.3” (see also Figure S2). To estimate the probability of infection with each lineage given that the cohort‐site was tested and genotyped (parameters *ρ*_1_, *ρ*_2_, and *ρ*_3_), the model considered whether each of the three routes exposed a cohort‐site to a lineage (with values 1 or 0 indicating that exposure was or was not possible, respectively); hence, a cohort‐site could be exposed a maximum of three times. We then estimated the probability of infection with each lineage given exposure using Bernoulli likelihoods and vague priors (Beta(1, 1)).

### 2.6 Host model

In the host model, we tested whether the probability of infection for a given host was higher when exposed to IHNV by the same host or a different host type without considering the IHNV lineage identity. We focused on three host types: Chinook salmon, steelhead trout, and rainbow trout. Steelhead and rainbow trout are naturally distinct life‐history phenotypes of the same species, *O. mykiss*, where the former follows an anadromous life cycle involving long‐distance freshwater migration and a marine growth phase prior to return migration for spawning, whereas rainbow trout reside entirely in freshwater streams and lakes. We consider these life‐history forms as separate host types because of differential management practices in hatchery programs. In this model, we expect that lineage specialization should translate to higher probabilities of infection when exposure occurs by the same hosts compared to when exposure occurs by different host types. This is because specialized lineages would occur more frequently in the host type in which the lineage is adapted. In the absence of specialization, IHNV lineages should occur with equal frequency across hosts, so the probabilities of infection would be equivalent between exposing hosts.

Although the samples tested for IHNV were always identified with the salmonid host, if the host was not tested at the cohort‐site, there was no record of the host being present at the cohort‐site, making it difficult to determine whether the absence of a record was due to the host being absent from the cohort‐site or to a lack of testing. To distinguish between a host presence/absence versus a lack of IHNV testing in the cohort‐sites, we made the following assumptions. Juveniles in cohort *t* were assumed to be present at the site if adults of the same host type (i.e., progenitors) were tested in cohort *t* − 1. Similarly, adults were assumed to be present at the site in cohort *t* if juveniles of the same host type were tested at the site in cohort *t* + 1. If no records for either adults or juveniles were found in cohort *t* or *t* + 1, respectively, we assumed that the host type was absent from both cohorts of the site (see Figure S3 for a summary of host presence across cohort‐sites that includes the effects of this assumption).

Under this model, a cohort‐site could have been exposed to IHNV a maximum of six times (i.e., if a cohort‐site had the three hosts and each was exposed by the same host or other host types). Thus, the probability of infection of host *s* given IHNV exposure *P*(*I_s_*|*E*) was calculated as:

$$P\left(I_{s}|E\right)=\left\{\begin{array}{cc} \phi_{s}\left(1-\theta_{s}\right), & \text{when}\;\text{exposed}\;\text{by}\;\text{the}\;\text{same}\;\text{host}\;\text{only};\\ (1-\phi_{s})\theta_{s}, & \text{when}\;\text{exposed}\;\text{by}\;\text{other}\;\text{host}\;\text{types}\;\text{only};\\ \phi_{s}-\phi_{s}\theta_{s}+\theta_{s}, & \text{when}\;\text{exposed}\;\text{by}\;\text{the}\;\text{same}\;\text{or}\;\text{other}\;\text{host}\;\text{types}. \end{array}\right.$$

Here, *s* is either Chinook salmon, steelhead trout, or rainbow trout; *ϕ* is the probability of infection when exposed by the same host; and *θ* is the probability of infection when exposed by other host types.

### 2.7 Host‐by‐lineage model

In the host‐by‐lineage model, we estimated the probability of infection for the three salmonid hosts with lineages MD and UC. We excluded lineage UP from this model because only one positive test for this lineage was recorded among all cohort‐sites, and including this lineage in the model would add 12 parameters, which would likely increase estimation uncertainty. We expect to see evidence for specialization of lineage MD on steelhead and rainbow trout by the probability of infection with MD being greater in these hosts compared to Chinook salmon. By contrast, we expect to see similar probabilities of infection with lineage UC across salmon hosts, which would be consistent with the generalist phenotype suspected for this lineage (Black et al., 2016; Breyta, Black, et al., 2016).

In this model, a cohort‐site could be exposed a maximum of 12 ways, for example, if the three hosts were exposed to the two lineages by the same and other host types. We thus calculated the probability of infection *P*(*I_s_*_,_ *_l_*|*E*) of salmon host *s* given exposure with lineage *l* as:

$$P\left(I_{s,l}|E\right)=\left\{\begin{array}{cc} \alpha_{s,l}\left(1-\beta_{s,l}\right), & \text{when}\;\text{exposed}\;\text{by}\;\text{the}\;\text{same}\;\text{host}\;\text{only};\\ (1-\alpha_{s,l})\beta_{s,l}, & \text{when}\;\text{exposed}\;\text{by}\;\text{other}\;\text{host}\;\text{types}\;\text{only};\\ \alpha_{s,l}-\alpha_{s,l}\beta_{s,l}+\beta_{s,l}, & \text{when}\;\text{exposed}\;\text{by}\;\text{the}\;\text{same}\;\text{or}\;\text{other}\;\text{host}\;\text{types}. \end{array}\right.$$

Here, *α_s_*_,_ *_l_* is the probability of infection when exposed by the same host, and *β_s_*_,_ *_l_* is the probability of infection when exposed by other host types, such that *α_s_*_,_ *_l_β_s_*_,_ *_l_* is the probability of infection when exposed by the same and other host types.

## 3 RESULTS

### 3.1 Lineage model

Simulation results from the lineage model confirm that missing data have an effect on parameter estimation. Nevertheless, even with missing tests (i.e., Scenario 2; Figure S4) and missing tests and genotypes (i.e., Scenario 3; Figure S4) the true probabilities of infection with lineages MD and UC were always recovered (Figure S4), with the uncertainty of the parameter estimates extending approximately ±5% from the median estimates (Figure S4). We see a contrasting pattern for lineage UP. When the true probability of adult infection with this lineage was near‐zero (i.e., Cases 6, 7, and 8 in Figure S4), our simulations showed that the probability of juvenile infection with this lineage had high uncertainty, with 95% CIs approximately ±20% of the mean estimate.

**Figure 3:**
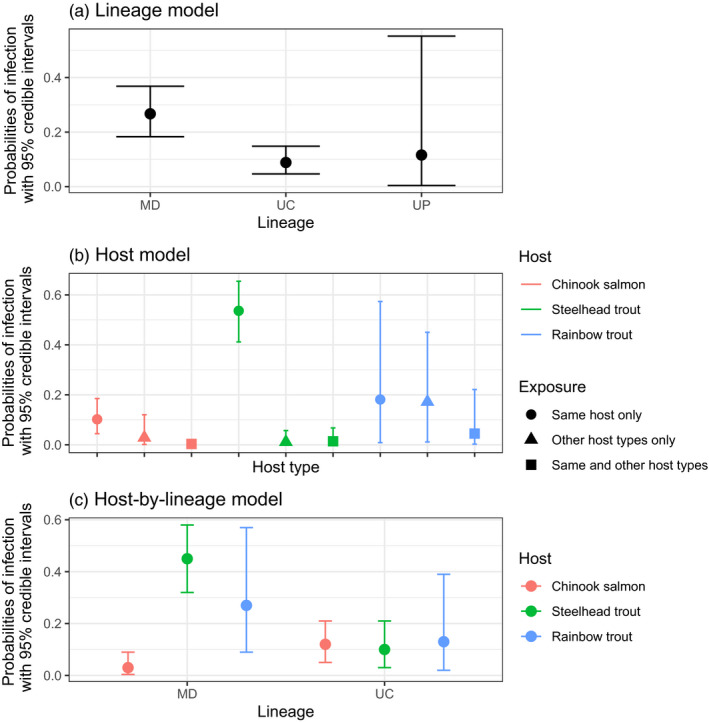
(a) Probabilities of juvenile infection with lineages MD, UC, and UP estimated from the lineage model. (b) Probabilities of juvenile infection estimated from the host model for Chinook salmon, steelhead trout, and rainbow trout (colors) when exposed to IHNV by the same host only, other host types only, or the same and other host types (while ignoring lineage identity). Notice that the probability of infection given exposure by the same or other host types is the sum of the three probabilities shown for a host type. (c) Overall probabilities of infection for the three host types with the two lineages estimated from the host‐by‐lineage model

When fitting the lineage model to the real data, we found that the probability of juvenile infection with lineage MD was higher (median estimate = 0.27) than the probability of infection with lineage UC (median estimate = 0.09), despite exposure to UC occurring most often (Figure 3a and Table 1). The probability of juvenile infection with lineage UP was characterized by wide CIs (Figure 3a) likely due to the fact that only one positive sample was observed for juveniles with this lineage. All additional estimates of model parameters are presented in Appendix S1 (Table S1).

**Table 1:** Summary of exposure events inferred by the lineage, host, and host‐by‐lineage models

Exposure class	Lineage	Host	Host‐by‐lineage
No. times exposed to MD only	30 (0.14)	—	196 (0.23)
No. times exposed to UC only	71 (0.32)	—	140 (0.17)
No. times exposed to MD and UC	120 (0.54)	—	486 (0.59)
No. times Chinook were exposed to any lineage	—	216 (0.33)	271 (0.33)
No. times steelhead were exposed to any lineage	—	216 (0.33)	292 (0.36)
No. times rainbow were exposed to any lineage	—	216 (0.33)	259 (0.31)
No. times Chinook exposed by Chinook only	—	49 (0.08)	44 (0.05)
No. times Chinook exposed by other only	—	52 (0.08)	80 (0.1)
No. times Chinook exposed by Chinook and other	—	115 (0.18)	147 (0.18)
No. times steelhead exposed by steelhead only	—	51 (0.08)	37 (0.05)
No. times steelhead exposed by other only	—	50 (0.08)	101 (0.12)
No. times steelhead exposed by steelhead and other	—	115 (0.18)	154 (0.19)
No. times rainbow exposed by rainbow only	—	0 (0.0)	3 (0.004)
No. times rainbow exposed by other only	—	186 (0.29)	176 (0.21)
No. times rainbow exposed by rainbow and other	—	30 (0.05)	80 (0.1)
Total number of exposures	221	648	822

Note

Values in parentheses are proportions of the total exposures. In the lineage model, a cohort‐site could have a maximum of two exposures: MD and UC (here, we exclude counts for lineage UP). In the host model, there was a maximum of six exposures if three hosts were present (3 hosts × 2 exposure sources). In the host‐by‐lineage model, each host could be exposed to lineage MD or UC by the same or other host type, so for each cohort‐site, a maximum of 12 exposures were possible (3 host types × 2 exposure sources × 2 lineages).

### 3.2 Host model

Simulation results showed evidence of underestimation for the probability of infection given exposure by other host types when the true value of the probability of infection given exposure by the same host was greater than approximately 0.5 (see Figure S5, Cases C1, C2, and C3). These biases were not, however, detected for lower probabilities of infection given exposure by the same host. Our simulation results also suggested that the probabilities of infection for rainbow trout were characterized by wider CIs compared to the other two hosts, which likely resulted from the lower number of hatcheries rearing rainbow trout (Figure S3) and the lower number of exposures to IHNV seen for this host type (Table 1).

Specific to the real data, the three studied hosts were frequently reared alone or with other host types so that exposure to IHNV by the same species only or by the same or other species was possible (Table 2). Estimates of the model fit showed that the probability of infection of juvenile steelhead trout was highest when exposure occurred by the same host (Figure 3b; see Table S1 for all estimates), even though steelhead trout had a similar number of exposures to IHNV by infected steelhead and other host types (Table 1). When exposed by the same host, steelhead trout had a higher probability of infection (median estimate = 0.54, 95% CI = 0.41, 0.66) than Chinook salmon (median estimate = 0.10, CI = 0.04, 0.19). For Chinook salmon, the probability of infection was similar for all exposing host types (Figure 3b).

**Table 2:** Frequency of different host types across cohort‐sites

	Chinook salmon	Steelhead trout	Rainbow trout
Single host	0.48 (76)	0.30 (38)	0.29 (11)
Co‐inhabiting with other host types	0.52 (82)	0.70 (89)	0.71 (27)
Overall presence of juveniles	0.51 (158)	0.41 (127)	0.12 (38)

Note

Single hosts indicate the frequency at which each host type was reared alone, whereas co‐inhabiting with other host types indicates the frequency at which hosts were reared with at least one other host type. Numbers in parentheses indicate the number of cohort‐sites in which hosts were found of a total of 312 cohort‐sites.

For juvenile rainbow trout, all estimates had high uncertainty as characterized by wide CIs (Figure 3b). Juvenile rainbow trout were observed only in 12% of the cohort‐sites (compared to 51% and 41% of the cohort‐sites for Chinook salmon and steelhead trout, respectively; Table 2, Figure S3), and the exposure history showed that exposure always involved other host types (Table 1). Thus, the high uncertainty in parameter estimation for rainbow trout was likely due to small sample sizes.

### 3.3 Host‐by‐lineage model

Simulation results suggested lower estimation precision in this model, as wide 95% CIs were associated with the parameter estimates. Nevertheless, simulations also suggested adequate estimation accuracy because median estimates were within ±20% of true parameter values. Greater estimation accuracy and precision were observed for Chinook salmon and steelhead trout compared to rainbow trout (Scenario 3 in Figure S6). Similarly to the lineage model, the precision of parameter estimates decreased as missing data increased due to no testing and both no testing and no genotyping.

In the model fit to the real data, we found that across all combinations of exposing host types (Figure 3c), the probability of infection with lineage MD was highest for steelhead trout (median estimate = 0.45, CI = 0.32, 0.58), followed by rainbow trout (median estimate = 0.27, CI = 0.09, 0.57), but was lowest for Chinook salmon (median estimate = 0.03, CI = 0.004, 0.09; Figure 3c). By contrast, across all combinations of exposing host types, the probability of infection with lineage UC was similar across the three host types (Chinook salmon median estimate = 0.12, CI = 0.05, 0.21; steelhead trout median estimate = 0.1, CI = 0.03, 0.21; rainbow trout median estimate UC = 0.13, CI = 0.02, 0.39; Figure 3c).

**Figure 4:**
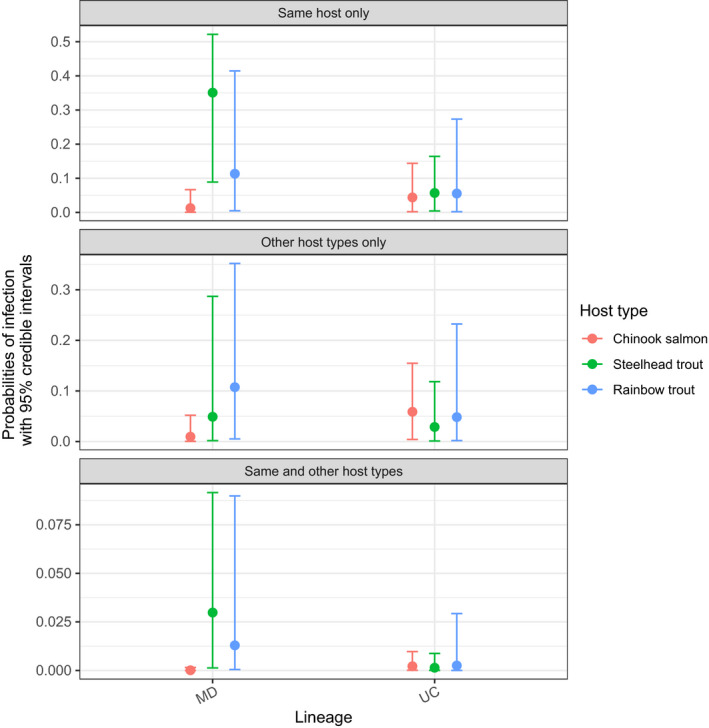
Probabilities of infection estimated by the host‐by‐lineage model specific to the exposing host types. The estimates are for probabilities of infection of juvenile Chinook salmon, steelhead trout, and rainbow trout with lineages MD and UC given exposure by the same host type only, other host types only, and the same and other host types

The contrast in infection probabilities between MD and UC is also evident when the exposing host type is considered (Figure 4). Specifically, we found that when exposed to lineage MD by the same host type, steelhead trout had a higher probability of infection than Chinook salmon (green and pink dots, top panel in Figure 4, median estimate for steelhead trout = 0.35, CI = 0.09, 0.52; median estimate for Chinook salmon = 0.01, CI = 0.0004, 0.07), despite exposure frequency to lineage MD being lower than to lineage UC in the former host (Table 1). Furthermore, when steelhead trout were exposed by infected steelhead, the probability of infection with lineage MD appeared higher than the probability of infection with lineage UC (green dots in the top panel, Figure 4), even when more exposures to lineage UC occurred compared to lineage MD (Table 1).

When exposed to lineage UC by the same host type, we found low probabilities of infection across the three host types (top panel, Figure 4), even though hosts were frequently exposed to this lineage (Table 1). All other comparisons between the lineages or across exposure sources (i.e., when exposed by the same host vs. when exposed by other host types) were characterized by overlapping CIs (Figure 4). However, a trend consistent with the hypothesis of trade‐offs associated with specialization is that the median estimates across all hosts for lineage UC (both when exposed by the same host only and when exposed by other host only) were higher than the median estimates for lineage MD in Chinook salmon.

## 4 DISCUSSION

Viral pathogens have ubiquitously evolved in ecological communities, but there are few systems where existing data describe the degree to which pathogens have specialized. Here, we use long‐term data on exposure and transmission to better characterize the ecological effects of pathogen specialization. In addition to quantifying probabilities of infection of socially and economically important salmonid hosts with different IHNV lineages, our results provide insights into the trade‐offs that viral lineages incur when multiple hosts are available. Our results suggest that the management of diseases in hatcheries can benefit from knowledge of the circulating viral lineages and of the infection risks that they pose for the reared host types.

Results from the lineage model suggested that lineage MD had a significantly higher probability of infecting juvenile fish than lineage UC across the cohort‐sites. Results from the host model then suggested that juvenile steelhead trout had a high probability of infection given exposure to other infected steelhead trout, even when ignoring the viral lineage identity. However, for the other host types, the exposing host had no detectable effect on the probability of infection (in rainbow trout, these comparisons were hampered by estimates with high uncertainty due to limited data). Finally, the host‐by‐lineage model showed that lineage MD appears specialized on steelhead and rainbow trout (both *O. mykiss*), because of high probabilities of infection given exposure, and not adapted to Chinook salmon, because of near‐zero probabilities of infection given exposure. Furthermore, in steelhead trout, we found that the probability of infection with lineage MD was highest when infected steelhead trout were the source of exposure, as opposed to other host types (Figure 4). In contrast to the specialist phenotype observed for lineage MD, lineage UC displayed a generalist phenotype because of its low probability of infection across host types (Figure 4). In all models, we also found that the probabilities of infection with lineages MD and UC were independent of the number of exposure events, as this number was similar for the two lineages. Thus, our results suggest that specialist–generalist strategies of IHNV lineages guide the infection dynamics of salmonid hosts in the Columbia River Basin.

Furthermore, our results provide an indication of the trade‐offs associated with these infection strategies at the landscape level (Remold, 2012; Woolhouse et al., 2001). The high probability of *O. mykiss* infection with lineage MD could be explained by specific viral adaptations resulting in a high rate of in‐host replication and/or transmission when exposed to this host. The specialization of lineage MD to *O. mykiss*, however, could come at a cost of low replication and/or transmission in other hosts, as seen by the near‐zero probability of infection of lineage MD in Chinook salmon. By contrast, the modest, but constant, probability of infection of lineage UC across host types could be explained by modest levels of replication/transmission across multiple hosts. While infecting multiple hosts can be advantageous, the hypothesized cost of generalism is that of constrained replication/transmission in any particular host type, although no‐cost generalism has also been observed in other systems (Remold, 2012; Woolhouse et al., 2001).

Overall, our results suggested that variation in host type composition across hatcheries can affect the incidence of IHNV lineages, likely because of specialist or generalist host–pathogen associations. Knowledge of viral lineage identity in hatcheries could, therefore, help managers adopt control measures that minimize IHNV exposure between similar host types (e.g., between adult and juvenile steelhead trout), which could lower juvenile mortality caused by high‐virulence specialized lineages compared to low‐virulence generalist lineages. As an example, successful reduction in juvenile mortality from infection with MD genotypes resulted from a management action that avoided exposure of juvenile steelhead trout to river water containing adult steelhead trout in the Dworshak hatchery (Idaho, 2009–2010) (Breyta, Samson, et al., 2016). Although this action also resulted in increased infection with UC lineages that were transmitted by adult Chinook salmon to juvenile steelhead trout, juvenile mortality was still reduced because the generalist UC lineage has low virulence in steelhead trout. In addition to managing water sources, more complex rearing strategies that minimize the overlap between adult and juveniles of the same species or that maximize juvenile species heterogeneity could have positive effects on the control of specialized lineages. Careful assessment of such management practices is, however, necessary because IHNV lineages can diversify rapidly (Kurath et al., 2003; Troyer et al., 2000) and shifting management practices that provide a competitive advantage of one lineage over others could set ideal conditions for the evolution of new host specialization.

Future experiments that quantify transmission rates under different combinations of IHNV lineages and host species could provide additional insights into the role that different host types play in transmission dynamics. In addition, evaluating the effects of pathogen evolution under single‐host and multihost configurations (Gandon, 2004; Rigaud et al., 2010) could characterize the role that the host community plays in lineage specialism/generalism. Additional work is also required to estimate hatchery‐specific probabilities of infection with IHNV lineages. In our models, probabilities of infection were inferred across all hatcheries. While our model simulations suggested that this resulted in robust parameter estimates, this procedure ignored hatchery‐specific effects that may influence the local incidence of different lineages.

Work that expands these analyses to include data collected for hatcheries in the upper Columbia River Basin and the Snake River Basin could also increase the precision of parameter estimates, such as the probability of infection with lineage UP and the probability of rainbow trout infection. Here, estimates of the probability of infection with lineage UP were imprecise because of the low incidence of lineage UP among the studied sites (only one positive detection in the 312 juvenile cohort‐sites). Thus, expanding these modeling efforts to include hatcheries along the Snake River where lineage UP and sockeye salmon are more prevalent may allow for precisely quantifying probabilities of infection with lineage UP. Similarly, we were unable to make precise inferences about the probability of infection of rainbow trout due to data limitations. In our study area, fewer hatcheries reared rainbow trout (10 out of 24 sites compared with 18–19 out of the 24 sites for either steelhead trout or Chinook salmon), and they were nearly always reared with other species. In addition, hatchery production of rainbow is aimed at restocking inland lakes for recreational fishing. This removes the effect of adult‐to‐juvenile exposure because adult rainbow trout do not migrate back to the hatchery. Additional data across more hatcheries may thus be needed to precisely estimate the probability of infection of this host type.

Assessing the risks that diseases impose on fish is an important step in preserving the health of managed wildlife. Previous studies have developed mechanistic SIR‐type models to propose mechanisms that explain disease dynamics in other fish host–pathogen/parasite systems (Boerlage, Graat, Verreth, & Jong, 2013; Carraro et al., 2016; Langwig et al., 2017; Turner, Smith, & Ridenhour, 2014). Such modeling efforts have provided insights into the efficacy of different control programs and on the ecology and evolution of infectious diseases in fishes. Our results contribute to this knowledge by elucidating the role that specialization plays in determining the incidence of different pathogen lineages in multiple salmonid hosts. Although generalist and specialist pathogen strategies have been documented in several vertebrate species (Kubinak et al., 2013; McCoy et al., 2013; Zukal et al., 2014), few studies demonstrate how such specialization affects the infection rates of coexisting vertebrate host life‐history types over broad geographic areas. Our results therefore demonstrate the usefulness of combining long‐term ecological data with statistical models to study the ecology and evolution of host–pathogen interactions.

## CONFLICT OF INTEREST

None declared.

## Data Availability

The data that support the findings of this study are openly available in Ecology Data Papers at https://doi.org/10.1002/ecy.1634 (Breyta, Brito, Kurath, et al., 2017).

## Appendix 1

**Table A1:** Description of the parameters estimated in the three models

Parameter	Description
Used in all models	
*λ*	Probability of first‐cohort juvenile infection
*η* ^a^	Probability of adult infection given no test
*ϵ* ^b^	Probability of juvenile infection given no test
Used in the lineage and host‐by‐lineage models	
*τ*_1_^c^, *τ*_2_^d^	Probabilities of adult infection with lineages MD and UC, respectively, given no genotyping of positive tests
*µ*_1_^e^, *µ*_2_^f^	Probabilities of juvenile infection with lineages MD and UC, respectively, given no genotyping of positive tests
*ν*_1_, *ν*_2_	Probabilities of adult infection with lineages MD and UC, respectively
Used in the lineage model only	
*τ*_3_^g^	Probability of adult infection with lineage UP given no genotyping of positive test
*µ*_3_^h^	Probability of juvenile infection with lineage UP given no genotyping of positive test
*ν*_3_	Probability of adult infection with lineage UP
*ρ*_1_, *ρ*_2_, *ρ*_3_	Probabilities of juvenile infection with lineages MD, UC, and UP, respectively
Used in the host model only	
*ν*_0_	Probability of adult infection
*ϕ*_1_, *ϕ*_2_, *ϕ*_3_	Probabilities of infection for juvenile Chinook salmon, steelhead trout, or rainbow trout, given exposure by the same host
*θ*_1_, *θ*_2_, *θ*_3_	Probabilities of infection for juvenile Chinook salmon, steelhead trout, or rainbow trout given exposure by other host types
Used in the host‐by‐lineage model only	
*α*_1,1_, *α*_2,1_, *α*_3,1_	Probability of juvenile infection with lineage MD given exposure by the same host (for all parameters here, the first subscript is the salmon host type: 1 = Chinook salmon, 2 = steelhead trout, and 3 = rainbow trout; and the second subscript is the lineage: 1 = MD and 2 = UC)
*α*_1,2_, *α*_2,2_, *α*_3,2_	Probability of juvenile infection with lineage UC given exposure by the same host
*β*_1,1_, *β*_2,1_, *β*_3,1_	Probability of juvenile infection with lineage MD given exposure by other host types
*β*_1,2_, *β*_2,2_, *β*_3,2_	Probability of juvenile infection with lineage UC given exposure by other host types

Note

Vague beta prior distributions (Beta(1, 1)) were used for all parameters without a superscript. Prior distributions for parameters with superscripts were specified as follows: a = Beta(4, 86), b = Beta(3, 182), c = Beta(39.52, 43.48), d = Beta(51.38, 31.62), e = Beta(26.25, 8.75), f = Beta(9.72, 25.28), g = Beta(0.83, 82.17), and h = Beta(0.97, 34.03). Ferguson et al. (2018) for priors a and b, and Breyta, Brito, Kurath, et al. (2017) for priors c–h.
