# Supplementary figures and images for: Infectious hematopoietic necrosis virus specialization in a multihost salmonid system
Source: Evol Appl. 2020 Feb 28;13(8):1841–53. doi: 10.1111/eva.12931 (PMC7463311; doi:10.1111/eva.12931)

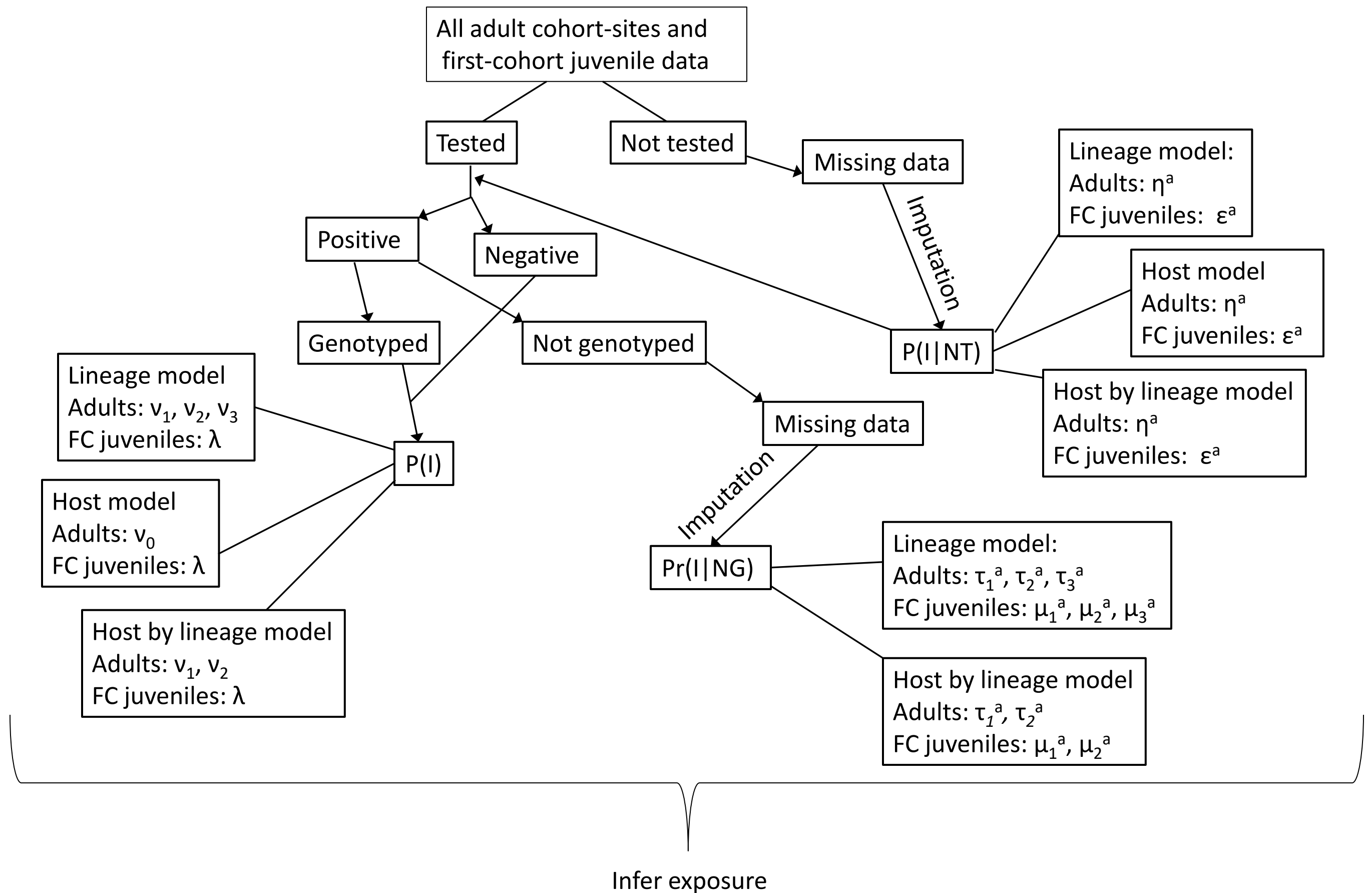

Supplement: Supplementary file 1 [file EVA-13-1841-s001.pdf]

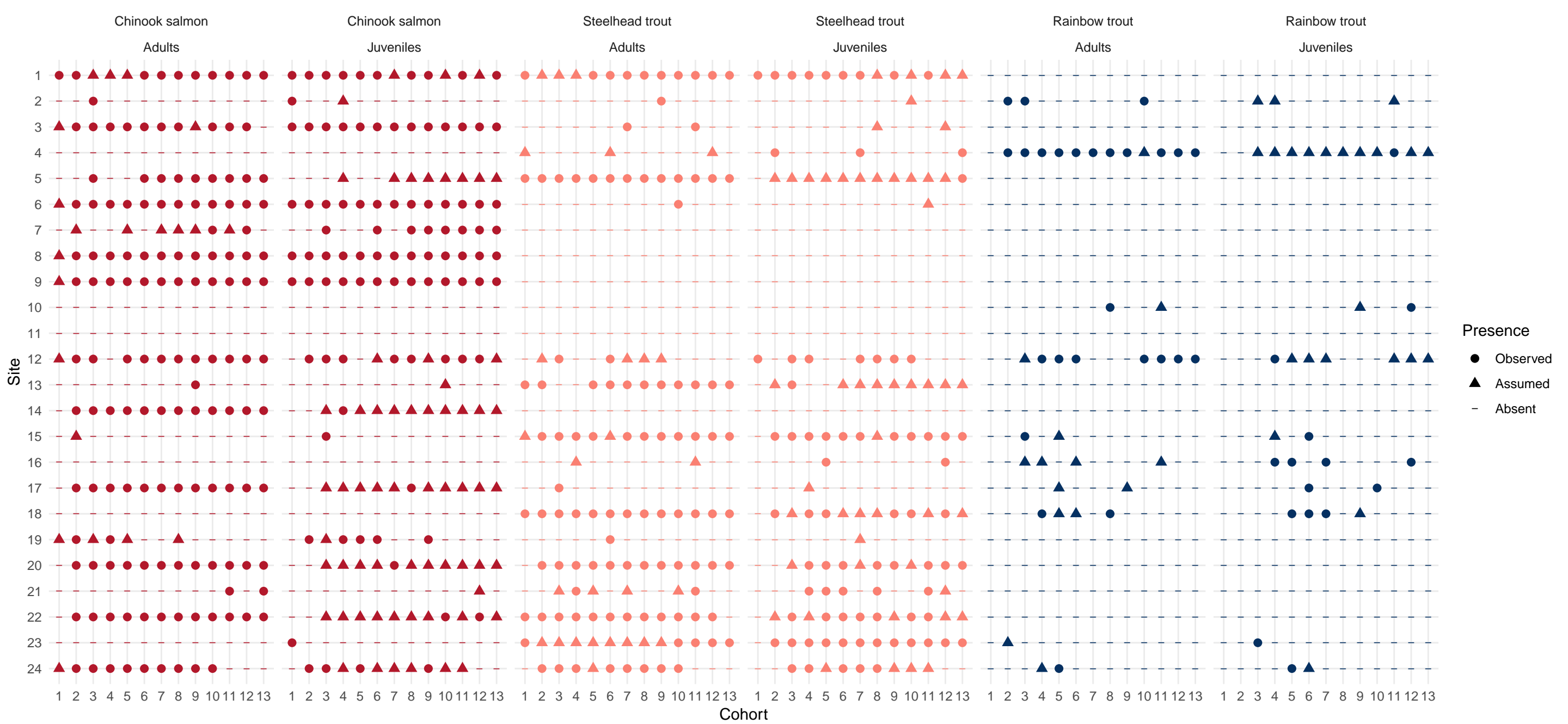

Supplement: Supplementary file 3 [file EVA-13-1841-s003.pdf]

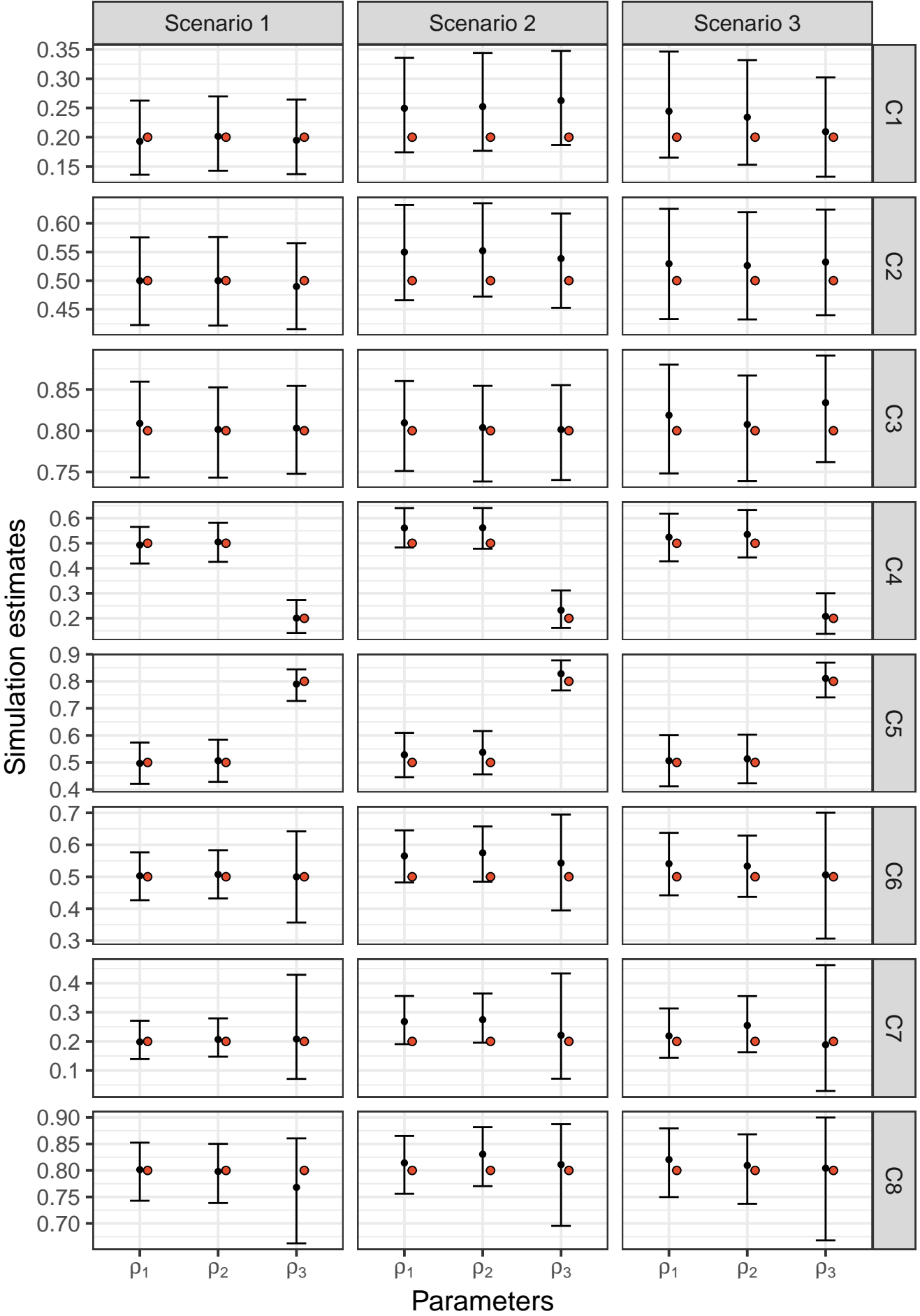

Supplement: Supplementary file 4 [file EVA-13-1841-s004.pdf]

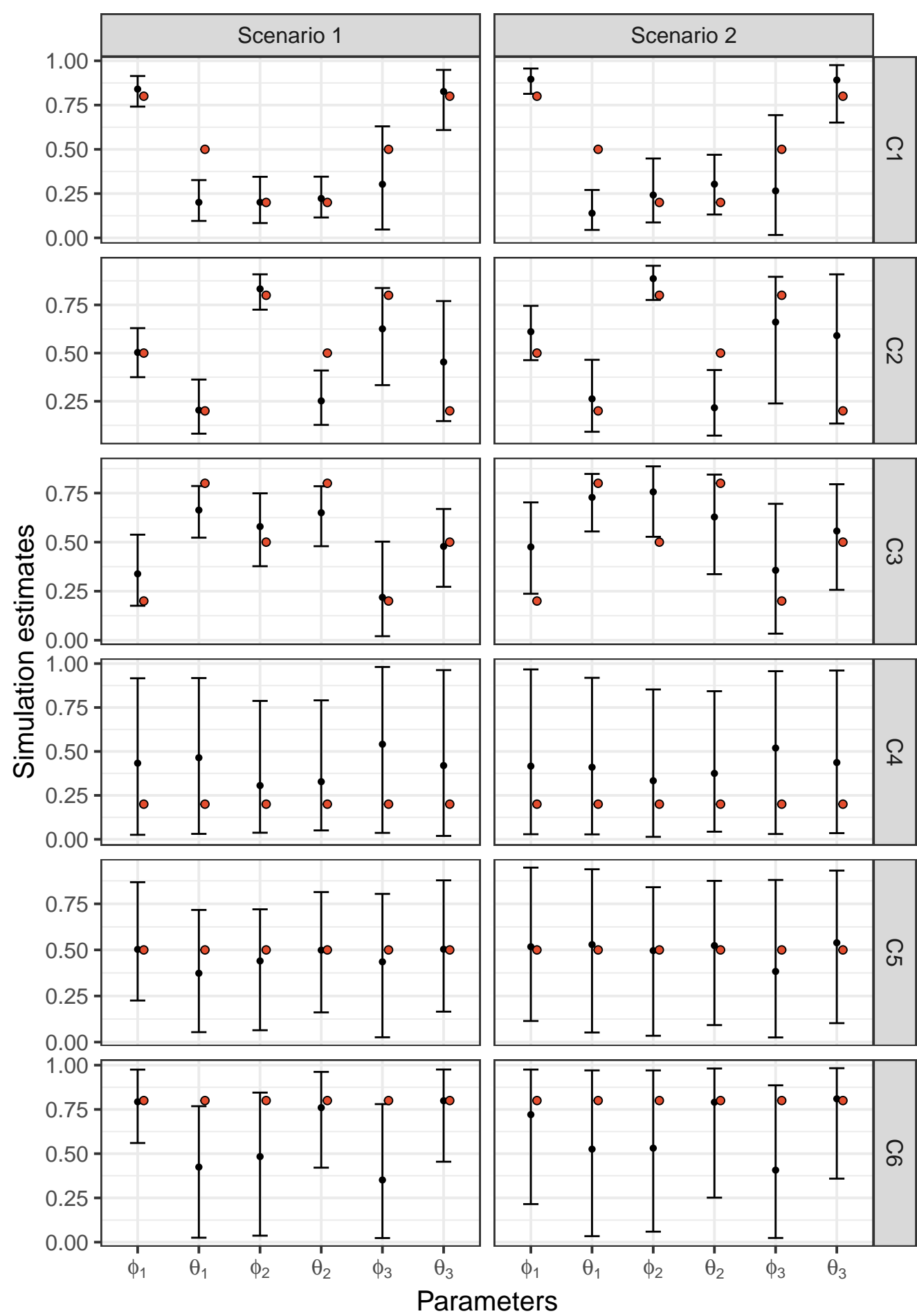

Supplement: Supplementary file 5 [file EVA-13-1841-s005.pdf]

Simulation estimates

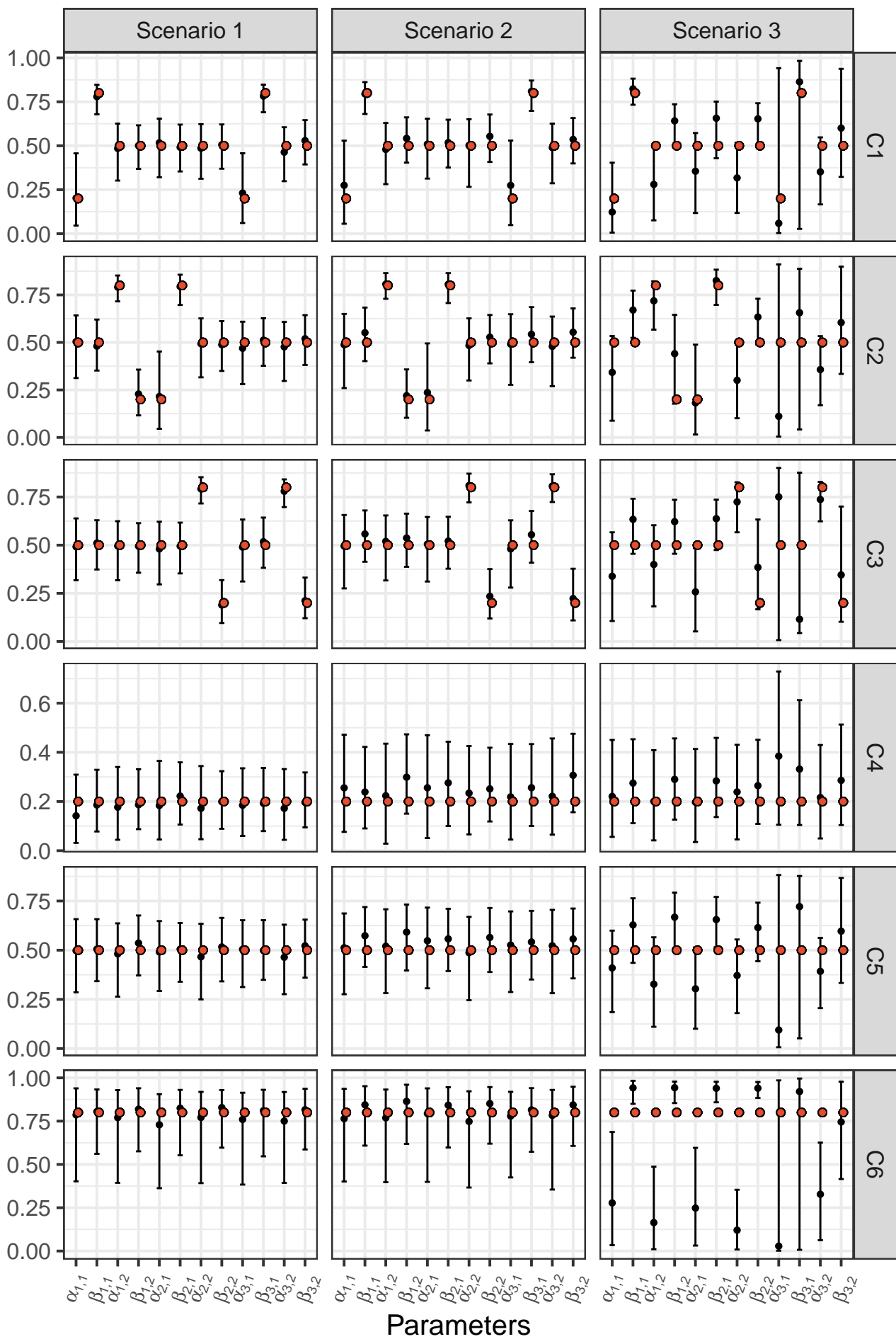

Supplement: Supplementary file 6 [file EVA-13-1841-s006.pdf]
