# Supplementary material for: Infectious hematopoietic necrosis virus specialization in a multihost salmonid system
Source: Evol Appl. 2020 Feb 28;13(8):1841–53. doi: 10.1111/eva.12931 (PMC7463311; doi:10.1111/eva.12931)
Supplement: Supplementary file 2 [file EVA-13-1841-s002.pdf]

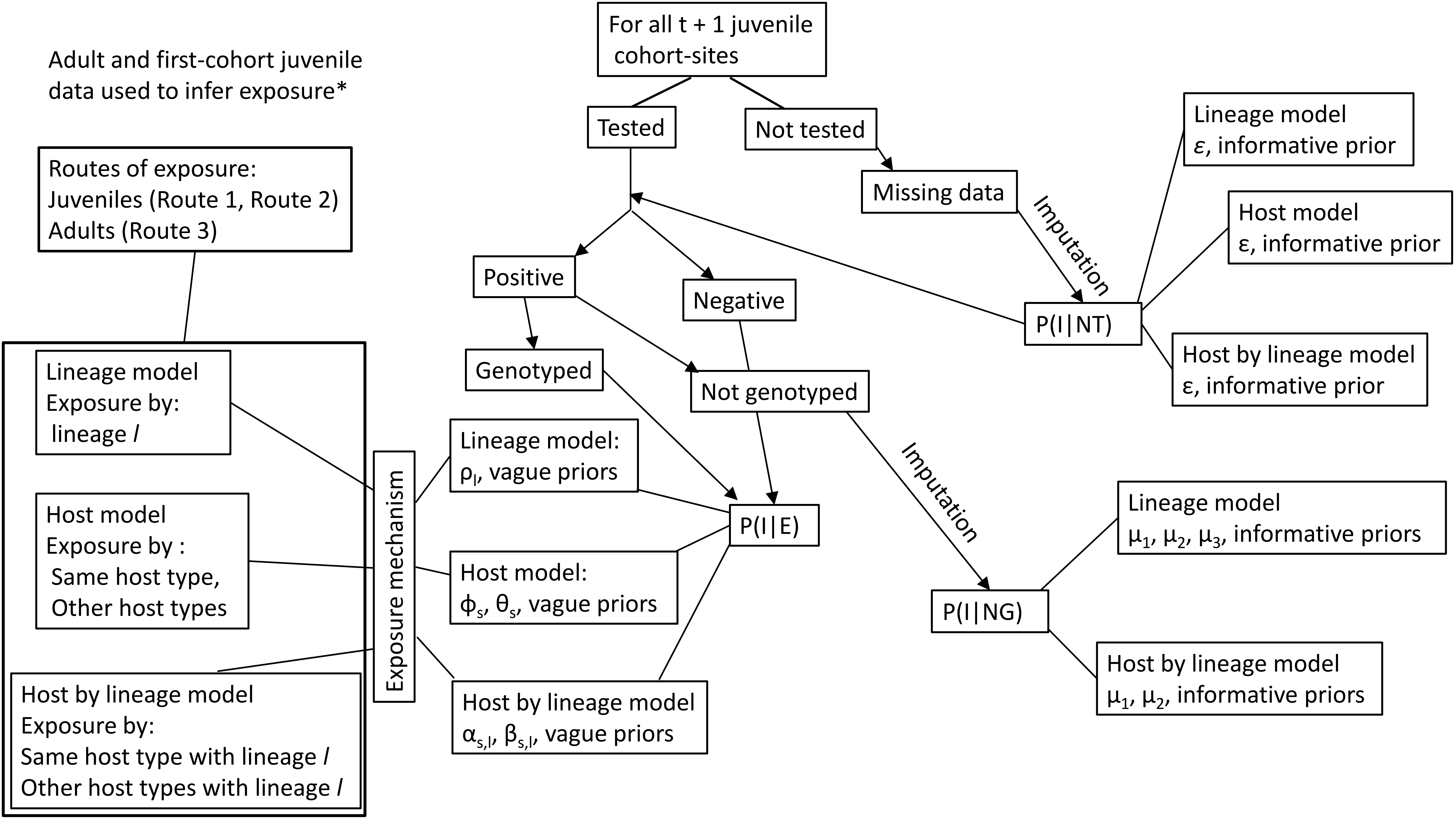

Adult and first-cohort juvenile data used to infer exposure*
Routes of exposure: Juveniles (Route 1, Route 2) Adults (Route 3)
Lineage model Exposure by: lineage *l*
Host model Exposure by : Same host type, Other host types
Host by lineage model Exposure by: Same host type with lineage *l* Other host types with lineage *l*
Exposure mechanism
For all t + 1 juvenile cohort-sites
Tested
Not tested
Missing data
Imputation
Positive
Negative
Genotyped
Not genotyped
Lineage model: ρ_l, vague priors
Host model: ϕ_s, θ_s, vague priors
Host by lineage model $\alpha_{s,l}$, $\beta_{s,l}$, vague priors
P(I|E)
P(I|NT)
Lineage model ε, informative prior
Host model ε, informative prior
Host by lineage model ε, informative prior
Imputation
P(I|NG)
Lineage model μ_1, μ_2, μ_3, informative priors
Host by lineage model μ_1, μ_2, informative priors
